# Partnership between staff and family in long-term care facility: a hybrid concept analysis

**DOI:** 10.1080/17482631.2020.1801179

**Published:** 2020-08-24

**Authors:** Hye-Young Jang

**Affiliations:** College of Nursing, Hanyang University, Seoul, Korea

**Keywords:** Concept analysis, partnership, family, staff, long-term care facility

## Abstract

**Purpose:**

The purpose of this study was to examine the attributes and verify the definition of the partnership concept using the hybrid model.

**Methods:**

A hybrid model was used to develop the concept of partnership. The hybrid model consists of three phases: theoretical, fieldwork and final analytical. In the theoretical phase, a working definition of partnership was developed by an extensive review with 35 studies. The fieldwork phase comprised seven focused-group interviews with 35 participants consisted of 25 facility staff and 10 family caregivers in long-term care facilities. The final analytical phase compared and interpreted the findings from the first and second phases in order to clarify the concept of partnership.

**Results:**

The concept of partnership was found to have two dimensions: interpersonal and environmental dimensions. The seven attributes emerged from this study. They included relationship, information sharing, shared decision-making, professional competence, negotiation, involvement in care, shared responsibility.

**Conclusions:**

The partnership between family and staff in long-term care facilities was defined as an ongoing and dynamic process associated with interpersonal and environmental factors. Based on the results, it can be suggested that the development of a tool for measuring partnership and an effective program for enhancing to establish a collaborative relationship.

## Introduction

The number of older adults who require long-term care (LTC) has increased rapidly with the increase in the ageing population. Within a culture of care that prioritizes caring by family members at home, caregivers experience physical, emotional, and social problems and despair (Jang & Yi, 2017). Despite somewhat negative perception of LTC facilities (Kwon & Tae, 2014), this heavy care burden has led to a steady increase in the institutionalization of older person, and the number of residents in LTC facilities in Korea reached 345,000 in 2016 (National Health Insurance Corporation, 2017).

Admission older adults to a LTC facility does not signify the termination of family care. While families often expect the burden of caring to be reduced due to the older adults’ entry into the facility, the families still have the caregiving burden (Majerovitz, 2007) and are confused about the changing role of caregiving (Chang & Schneider, 2010; Kwon & Tae, 2014; Mast, 2013). In addition, the family still wants to maintain a meaningful relationship with the older adult even after entering the facility and to continue to involve in care of the older adult (Bauer et al., 2003; Hagen, 2001). The families of older adult residents may provide important information on the life history, habits, preferences, and care needs of residents (Robison et al., 2007; Utley-Smith et al., 2009), so the family’s involvement in care is crucial for the well-being of the older adult residents (Bauer, 2007). Therefore, the family of resident should be regarded as a partner who expresses the preferences and expectations of the resident and participates in the care, not the passive watchers (Choi & Bang, 2013).

Since the concept of partnership was declared in 1978 by the World Health Organization as a key element of the goals for all people’s health (World Health Organization, 1978), it focuses on improving the health status and health-care ability of the patients, and is used in cooperation with the health-care providers (Choi & Bang, 2013).

In Western countries, since Casey (1988) proposed the partnership nursing model, research on partnerships not only in hospitals but also in community practice has been actively conducted, noting that collaborative relationships between health-care providers and patients have a positive impact on patients’ health (Lee, 1998). Previous researches on partnerships included exploring the meaning of partnerships and analysing concepts such as negotiation, equality of care, involvement in care (Casey, 1995; Dowling et al., 2004; Espezel & Canam, 2003; Lee, 2007), partnership models (Courtney et al., 1996; Farrell, 1992), and family involvement (Coyne & Cowley, 2007; Power & Frank, 2008). In addition, family involvement in care in facility has a positive effect on the older adult, the family and the facility staff (Pillemer et al., 2003; Robison et al., 2007) and is an important factor in ensuring the quality of life of the older adult resident. Various nursing interventions are applied to prevent role conflicts and to build cooperative relationships among the family members and staff members (Specht et al., 2000) and the concept of partnership between them is emphasized (Haesler et al., 2010).

Despite the necessity and importance of partnerships are widely known and emphasized abroad, studies on partnerships are insufficient in Korea. Most are limited to exploratory research on partnerships in hospital settings for nurses and families of hospitalized children (Bae & Lee, 2017; Choi & Kim, 2014). Research on this has not been actively conducted in various fields such as long-term care facilities and community. On the other hand, there is a study that conducted a concept analysis as a part of the tool development process for the partnership among the parents of hospitalized children and nurses in Korea (Choi & Bang, 2013). However, there are limitations in applying the results to the formation of partnerships with residents’ families and facility staff.

In other words, in a hospital setting, the family stays as a guardian for a limited length of stay and participates in the treatment and care of the patient. In contrast, in a facility setting, the family does not reside during their stay, but rather visits the facility and participates in caring for the resident. So the partnerships with families and health-care providers in hospital and institutional settings can be seen as being formed through different dynamics in different contexts. Therefore, in order to have a clear understanding of the partnership of the family and staff in the facility, it is necessary to consider the context that affects the formation of the partnership. Applying the meaning of the concept used in the existing literature as it may not be able to reflect the actual situation of the facility.

In order to promote positive outcomes in the health status of the older adults in the facility and to increase the family’s adaptation to daily life after entering the facility, various efforts are needed to form a partnership between the family and the facility staff. First of all, the establishment of a conceptual definition of the partnership between staff member and family member is essential. This is not only a basic data for intervention program and policy development for partnership building but also a direct contribution to the development of tools that can assess the partnership between resident’s families and staff member.

Therefore, the purpose of this study was to examine the attributes and verify the definition of the partnership concept between staff members and residents’ family members in LTC facilities, using the hybrid model (Schwartz-Barcott & Kim, 2000).

## Methods

### Study design

This study was performed to concept analysis with a hybrid model by Schwartz-Barcott and Kim (2000) that determine the dimensions, attributes, and indicators of partnerships between staff members and residents’ family members in LTC facilities.

### Study procedure

**Figure 1.**
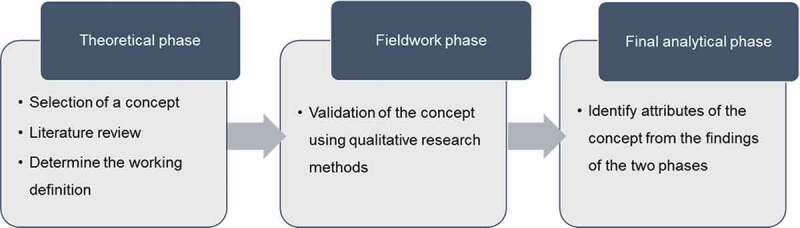
Hybrid model of concept analysis of partnership.

To develop a concept of partnership in LTC facilities, this study was performed to analysis using the hybrid model (Schwartz-Barcott & Kim, 2000). This model is a way to create, develop, and expand concepts, especially widely used to clarify of concept in the field of nursing. The hybrid model combines the inductive and deductive approaches and integrates theoretical analysis with empirical investigations. This model comprises three phases: theoretical, fieldwork, and final analytical phase (Figure 1). The theoretical phase begins with the selection of a concept of interest in the field of practice. Then, the literature is searched and reviewed to formulate the working definition. The fieldwork phase is undertaken to verify the concept of empirically using qualitative methods. The final analytical phase consists of a conceptual analysis of the findings from the two phases to identify attributes of the concept. Through this model, the concept is refined and new and more comprehensive definitions emerged, and at times quite different definitions from the initial ones (Schwartz-Barcott & Kim, 2000).

#### Theoretical phase

**Figure 2.**
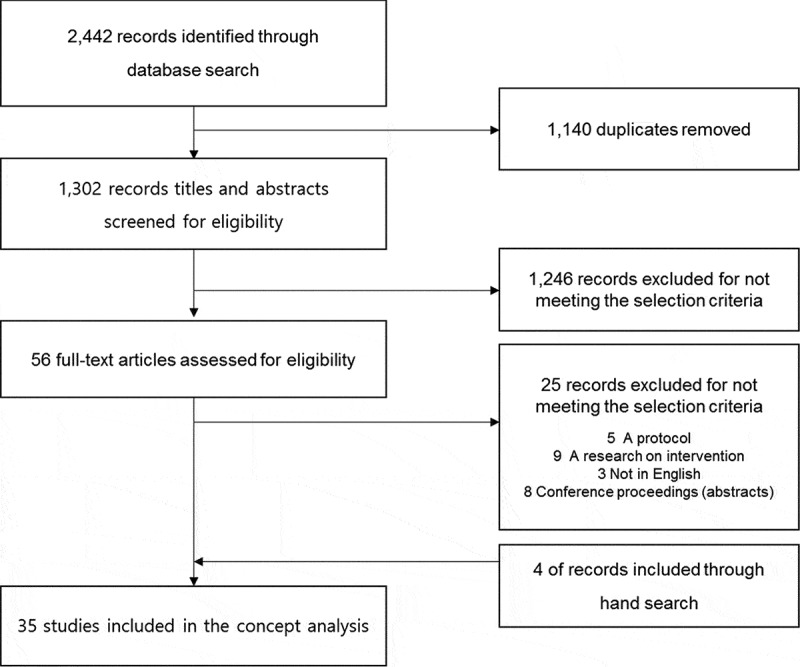
Flowchart of literature search and selection.

For a theoretical analysis of partnership in LTC facilities, the literature was systematic reviewed. Search terms used were (famil* OR staff*) AND (partnership OR partner OR collaboration OR cooperation) AND (“nursing homes” OR “long-term care facility”) were searched. A search was performed in these databases; Korean articles in the KoreaMed, KMbase, Research Information Sharing Service (RISS), Koreanstudies Information Service System (KISS), and National Digital Science Library (NDSL), and foreign articles in the PubMed, Excerpta Medica dataBASE (EMBASE), PsycINFO, Cumulative Index to Nursing and Allied Health Literature (CINAHL) database, and Cochrane Library. The articles published from 1980 to 2016 were included in the search, based on the previous study (Gallant et al., 2002) that considered the 1980s as the period during which the concept of partnership in nurse-client emerged. Only articles written in English and Korean. Two researchers undertook the literature search independently and yielded 2,442 articles; after duplicates were removed, 1,302 articles were left for review. Following a review of titles and abstracts, 1,246 articles determined to be unrelated to the topic were excluded, remaining 56 articles. The full text of these articles was reviewed, and 25 articles were excluded for not meeting the selection criteria. Four additional hand-searched articles identified during the process of reviewing the articles’ full text were included. Finally, 35 articles were included in this study (Figure 2).

The articles were analysed systematically to determine the working definition and attributes of the partnership in LTC facilities. The antecedents, attributes, and consequences presented in each article are as shown in Table I.

**Table I.** Antecedents, Attributes and Consequences of partnership in Literature Review.

Authors	Antecedents	Attributes	Consequences
Dupuis et al., 2016	None	Connecting and committing, creating a safe space, valuing diverse perspectives, establishing and maintaining open communication, conducting regular critical reflection and dialogue	Resident: improved equality
Bauer et al., 2014	Staff attitudes, mutual cooperation, meaningful engagement, shared expectation	Building trust, involvement, keeping the family happy	Resident: maintaining the health and well-being Family: satisfaction of facility Staff: having confidence to provide good care
Choi & Bang, 2013	None	Reciprocity, professional knowledge & skill, sensitivity, collaboration, communication, shared information, cautiousness	None
Park & Jang, 2010	Mutual respect	Sharing information, sharing power, autonomy, sharing decision making	Resident: enhance participant`s adherence, health status, and the quality of life
Cowdell, 2009	None	Sharing information, sharing the care, developing supportive relationships, making it work	
Haswell-Elkins et al., 2009	None	Gaining two-way understanding, supporting the empowerment	Achieving greater wellness
Utley-Smith et al., 2009	None	Interaction, communication	Improved quality of care for residents
McVeigh et al., 2009	None	Opening lines of communication, acknowledging, providing support	Family: improved satisfaction of facility
Alice Lau et al., 2008	Beliefs, experiences about institutionalization, role relationship expectation	Institutional social penetration: self-disclosure, evaluation of care, penetration strategies	Resident: no resistance towards institutionalization
Wiggins, 2008	Shared value, skill in relationships and communication, interpersonal skill, the presence of support, sharing and a conductive environment	Shared responsibility, information, decision making, communication, trust, respect, reciprocity	Patients, family, physician, nurse: positively impact on safety, quality of care, satisfaction, outcomes and job fulfilment
Haesler et al., 2006	None	Collaboration, positive communication, sharing information, sharing power and control	Increasing family involvement in resident care
Hook, 2006	Professional staff: values, knowledge and skills in relationship building, communication, clinical competence, introspection Environment: safe, time, leadership support, interdisciplinary relationship	Professional competency, communication, patient participation, relationship, shared knowledge, shared power, patient autonomy, shared decision-making	Empowerment: enhanced self-management, improved health care utilization, improved health outcomes
Bidmead & Cowley, 2005	Model of health visiting, organizational and professional support, practitioners` qualities and skills	A genuine and trusting relationship, honest and open communication and listening, praise and encouragement, reciprocity, empathy, sharing and respect for the other`s expertise, working together with negotiation of goals, plans, and boundaries, participation and involvement, support and advocacy, information giving, enabling choice and equity	Client: feel enabled and empowered, gain knowledge and self-esteem, change attitudes and behaviour Parents: perceive themselves as more capable, more supported, family relationship improved and child behaviour better Practitioner: more job satisfaction, less stress, greater role clarity
Blue-Banning et al., 2004	None	Communication, commitment, equality, skills, trusts, respect	Child: improved quality of life Professional: improved academic achievement and functional life skills
Bauer et al., 2003	Visiting nursing home	Establishing and maintenance of relationships, Be involved in care, collaborative with nursing home staff, be involved on decision-making processes, share in the responsibility of caring, shared understanding of responsibility for each task	Resident: adaptation of facility, psychosocial well-being Family: emotional support, social contact, relief of guilt, be satisfied with a resident’s care Staff: ameliorate the associated distress
Gallant et al., 2002	Democratic, value cooperation, commitment to shared responsibility, open and respectful, basic interpersonal skills	Structure: relationship Process: power sharing, negotiation	Empowerment
Gwyther, 2001	None	Relationship, involvement, communication	Adaptation of facility transition
Janzen, 2001	None	Monitoring care, communication, collaboration, relationship	Promote good quality care, acceptable quality of life for the resident
McQueen, 2000	None	Mutual and unilateral relationship, empathetic understanding, genuineness, unconditional positive regard	Patient focused care
Norris, 2000	None	Personal interactions, responsibility, mutual respect	Avoid conflict, provide high quality of care
Owen et al., 2000	None	Sharing information, seeking information, adult relations	Improved quality of care
Specht et al., 2000	None	Negotiation and involvement in care	Improved perception of caregiving role and knowledge of Alzheimers’
Lee, 1999	Belief, intention, adequate facility	Negotiation, equality of care between parents and nurses, involvement of families in care	Nurse’s role change in a supervisory role
Pillemer et al., 1998	None	Relationship, communication, cooperation, work together, understanding differences in values	Resident: improved quality of life Family-staff: reduce conflict
Friedemann et al., 1997	Extensive education of staff, the subsequent willingness of staff	Involvement, interactions	None
Courtney et al., 1996	None	Negotiated sharing of power, agree to be involved as active participants	Enhance the capacity of the partners
Leahey & Harper-Jaques, 1996	Beliefs, values	Reciprocity, non-hierarchical relationship, respect each as expert, aware of resourced and strengths, simultaneously feedback process	None
Taylor, 1996	None	Open lines of communication, participation, providing information	Parent: enabled to discuss their role and negotiate fully in the care of their child
Harvath et al., 1994	None	Blending local and cosmopolitan knowledge, unique information and nurses` knowledge and skills	Resident: tailored care, individualization care, improving care quality
Wade, 1995	None	Relationship, reciprocity, sharing, equality, respect, participation	empowerment
Farrell, 1992	Commitment of healthcare workers	Relationship of equality, share knowledge and teach the skill, acknowledge the unique of nursing care	Child: become more independent Family: have the responsibility for care
Stower, 1992	None	Family centred care, parent participation, negotiation, respecting the wishes	None
Opie, 1991	Recognition of the limits, organization of formal services, reorientation, focus on prevention services, integration of males into caring work	Equal relationship, sharing power and responsibility, participated in decision making	A cost saving
Casey, 1988	None	Relationship, negotiation, respect for the wishes of the family	Child: the child learn self-care until he is independent and considered mature
Teasdale, 1987	Change attitudes of nurse and patient	An equal relationship, involved in care, choice including negotiation, shared information	

#### Fieldwork phase

To confirm the attributes of the concept determined in the theoretical analytic phase, focus groups interviews were conducted with staff members and residents’ family members. The study participants consisted of individuals who could communicate without assistance and provided voluntary consent to participate in the study based on a full understanding of the study purpose. The detailed inclusion criteria were as follows:

• Staff who have worked at the current LTC facility for three or more months and were capable of adequately providing their experiences of working with residents and their family members.

• Resident’s family members were relatives of older adult who have resided in current facility for three or more months, were primary caregivers of their older adult, and visited them at the facility most frequently.

For the focus group interview, a researcher with extensive experience in qualitative studies drafted the interview questions based on the attributes of the concept as determined in the theoretical analytic phase. The interview questions were as follows: “What do you think is role of residents’ family in facility?,” “How do you feel about family members participating in residential care?,” “What do you think about the nursing home staff and residents’ family partnership?,” “What do you think is helpful (or necessary) when establishing partnership between staff and family?” and “What bothers you in establishing partnership between staff and family?”

Data were collected from May 2016 to August 2016 and the interviews were conducted in a quiet conference room or visiting room in facility and lasted approximately for 90 minutes. Data were collected until data saturation had occurred and no new information could be obtained. Finally, there were 35 participants (25 staff members in five groups and 10 family members in two groups). Of the staff participants, 24 were female, the average age was 52.8 years, and the average working period was 5.1 years. Of the family members participants, 7 were female, the average age was 52.6 years, and the average duration of institutionalization was 2.9 years. The general characteristics of participants are shown in Table II.

**Table II.** General Characteristics of Participants.

	Characters	Categories	n (%) or M± SD
Staff (N = 25)	Gender	Female Male	24 (96) 1 (4)
	Age (yr)		53 ± 8.4
	Education level	≤Middle school High school ≥College	2 (8) 11 (44) 12 (48)
	Work experience (yr)	<5 5–9 ≥10	5.1 ± 3.4 14 (56) 10 (40) 1 (4)
Family caregiver (N = 10)	Gender	Female Male	7 (70) 3 (30)
	Age (yr)		52.6 ± 9.0
	Education level	≤Middle school High school ≥ College	0 (0) 4 (40) 6 (60)
	Relationship to resident	Son Daughter Daughter-in-law	3(30) 4(40) 3(30)
	Duration of care before institutionalization (yr)	<1 1–5 6–9 ≥10	3.6 ± 1.0 2 (20) 5 (50) 2 (20) 1 (10)
	Duration of institutionalization (yr)	<1 1–4 ≥5	2.9 ± 2.7 3 (30) 5 (50) 2 (20)

To ensure the trustworthiness of data, in-depth interviews were conducted with one or two participants from each staff and family group, and we received feedback from peers to establish the validity of the analysis and interpretation. In addition, purposive sampling was used to facilitate transferability of the inquiry, and the interview data transcribed within 24 hours of finishing each interview to ensure that no data were missing or distorted (Anney, 2014; Lincoln & Guba, 1985).

The interviews were transcribed verbatim and analysed according to the qualitative content analysis using the qualitative computer software program ‘MAXQDA12ʹ(VERBI Software GmbH, Berlin, Germany). Qualitative content analysis is a research method that has been widely used to analyse the meaning of extensive and complex text-based data (Hsieh & Shannon, 2005). The details of this analytical procedure are as follows: First, coding was performed by repeatedly reading transcribed data to identify meaningful words, phrases, and sentences. Second, the codes are sorted into subcategories by comparing the differences and similarities between codes. Finally, subcategories are organized into categories depending on the relationships between subcategories.

#### Final analytical phase

In the final analytic phase, this study emerged the final attributes and definition of partnership in LTC facilities via comparing the findings through a literature review and the focus group interviews.

### Ethical considerations

Data were collected after obtaining approval from the Institutional Review Board at the researcher’s affiliated university (IRB No. HYI-16-036-2). Participants were explained the purpose of the study and were informed that the interviews would be recorded and that they could withdraw from the study at any time without negative consequences. Only those who voluntarily participated were asked to interview after giving written consent.

## Results

### Theoretical phase

#### Definition of partnership in the other academic field

A partnership is defined as a “relationship between individuals or groups that is characterized by mutual cooperation and responsibility, as for the achievement of a specified goal” (American Heritage Dictionary, 2006). Partnerships are prominent in a variety of fields, particularly economics, sociology, and education. In economics, partnership is described as the fundamental belief or assumption of primarily cooperative behaviour (Boardman & Vining, 2012). In the social sector, partners share a common vision, present opportunities to achieve multiple organizational benefits that include the development of a positive corporate culture and the opportunity to build reputational capital (McDonald, 2014). In the educational field, partnership is defined as comprehensive service activities that share various resources to achieve common goals (Cho & Kim, 2013).

#### Definition of partnership in nursing

In the nursing field, the concept of partnership has emerged since the World Health Organization (WHO) declared it as a key element of the goal to be achieved health for all individuals (World Health Organization, 1978). Traditionally, the relationship between health-care professionals and patients was hierarchical. Because health-care professionals have abundant knowledge about the patients’ diseases, it was common for health-care professionals to determine overall treatment procedures, and patients would comply with these established treatment plans. However, due to the social change that regards the patient as a health service consumer, the patients are perceived as actively managing their own health (McQueen, 2000). Moreover, health-care professionals, including nurses, have been viewed as care partners who enhance residents’ ability to manage their own health, rather than health-care providers or decision makers (Gallant et al., 2002). Accordingly, this shift in perspective regarding these roles has increased awareness of the importance of cooperative relationships between patients and health-care professionals. Consequently, the concept of partnership has been emphasized in the nursing field because collaboration with patients is perceived to be an important factor in improving patients’ health conditions and health-care abilities (Choi & Bang, 2013). In the nursing field, partnership is defined as the improvement of a patient’s healthcare ability through collaboration with the patient.

#### Partnership-related concepts

Concepts that are used interchangeably with partnership include alliance/therapeutic alliance; participation; empowerment; patient-, client-, or family-centred care; individualized care; patient involvement; physician–client relationship; interpersonal relationship; supportive relationship; and sharing. Of these, sharing, participation, and relationship considered as one of the attributes to explain the partnership (Bidmead & Cowley, 2005; Dupuis et al., 2016; Gallant et al., 2002; Hook, 2006). Addition, patient- and family-centred care emphasize family strengths and encourages family choice and control over decisions regarding services, while intervention effects based on family-centred care are assessed according to improvements in family members’ sense of personal control and self-efficacy (McCormack, 2004). Consequently, these concepts focus more closely on family empowerment than the interaction between family members and professionals. On the other hand, because empowerment entails development of the ability to take care of oneself, it could be considered an outcome of partnership, rather than a similar concept (Bidmead & Cowley, 2005; Choi & Bang, 2013; Gallant et al., 2002; Hook, 2006), and can, therefore, be distinguished from the concept of partnership.

#### Attributes of partnerships in LTC facilities

The attributes of partnership in LTC facilities were extracted seven elements from the literature review: relationship, information sharing, sharing of decision-making, professional competence, negotiation, involvement in care, and shared responsibility (Table III). As the first element, relationship is the most crucial and fundamental aspect of a partnership (Wiggins, 2008). Particularly, a trusted relationship not only allows family members to talk to nursing home staff about their concerns and fears of residents’ care but also facilitates the establishment of realistic care-related goals (Wiggins, 2008). As the second element, information sharing is based on the perceptions of both staff and family members as important resources in resident care. In other words, staff members possess professional nursing knowledge and clinical experience of elderly care, while family members provide unique information about their relative, such as habits, preferences, and care needs (Gallant et al., 2002; Robison et al., 2007). Therefore, information sharing through mutual interaction is a major attribute of partnership, and integrating nursing knowledge and individual patient information is required for effective and sensitive care (Holman & Lorig, 2000). Consequently, information sharing contributes to the provision of individualized and optimal care services for residents. The third element, shared decision-making is a process in which staff and family members find solutions together, rather than alone when faced with problems or decisions regarding the resident. Additionally, the family member is recognized as a partner playing an active rather than passive role in healthcare (McQueen, 2000). The fourth element, professional competence refers to professional knowledge and skills that can be applied to clinical practice (Wiggins, 2008). This includes the ability to provide care that meets individuals’ specific needs, identification of patients’ conditions, provision of appropriate responses, and the ability to provide education for patients’ empowerment. As the fifth element, negotiation refers to choosing care for resident when care plans are established and discussing the family roles in resident care with staff members (Hook, 2006). This is a premise that it is possible to negotiate goals, plans and scopes in providing care to the residents (Bidmead & Cowley, 2005). This can be clarified the expectations and roles of each other and helping family members to actively participate in decision-making (Choi & Bang, 2013). As the sixth element, involvement in care refers to the process in which both family and staff members serve as joint care providers in emotionally supporting and helping older adults to adapt to facilities (Coyne & Cowley, 2007). Since equal authority is required for effective involvement, trust in the ability of the other caregiving partner and mutual respect are fundamental (Gallant et al., 2002). As the final element, shared responsibility refers to sharing common caregiving goals and a sincere interest in the resident’s condition by both parties. Considering common goals as important and commitment to the resident are fundamental for active responsibility sharing.

**Table III.** Category, Subcategory, and Codes Obtained Among Staff in the Fieldwork Phase.

Category	Subcategory	Codes	A sample of participants’ statements
Relationship building through communication	Open communication	Open mind, Communication, Relationship	“If we have listen to first, a cooperative relationship naturally will be maintained”
	Building mutual understanding and trust	Trust relationship, Mutual understanding, Cooperation	“Cooperation is not achieved until trust is formed. I don’t believe each other here, and if I don’t understand, cooperation seems difficult. When trust builds, everything becomes easier from then on.”
Provision of information about the status of the resident	Need to be aware of the older adult’s condition	Not knowing the condition of the older adults, Not knowing the condition change, Lack of understanding of status change	“The relationship is well formed and understanding is improved only when they are aware of their parents’ status.”
	Provide opportunities for questions about the older adults’s condition	Check your questions, Encourage to ask questions, Creating a comfortable atmosphere	“They asked a question that they were interested in, but when they understood it, their facial expression certainly changed, and from then on, little change has come.”
Cooperative interaction in problem solving	Relationships seeking help from one another for the care of the older adults	Support, Mutual help, Close cooperation	“As long as older adult is here, we will seek help from his/her caregiver, and we will also ask he/she to help us.”
	Find solutions together through decision support	Decision support, Finding a solution	“We are grateful to those caregivers who gather together to discuss, think together, and seek solutions together when residents have a problem.”
Provision of high-quality care	Show with care rather than words	Respect for demands, Individualized care	“Basically, we have to be good at caring. I think showing them as care rather than words is the way to build trust.”
	Providing of safe care	Fall prevention, Fracture prevention, Injury prevention	“Older adults have fallen accidents. There may be safety accidents. When safety accidents happen, most of the trust you have built up so far is broken.”
Coordination of role and expectations at the facility	Lack of awareness of the role of the facility	Don’t know the role, The thinking that everything is possible here.	“A nursing home is not a hospital. Even if we have a nurse, you need to go to the hospital for treatment if necessary, but the family should do to us at the nursing home. This is how it’s like this.”
	Excessive expectations for caring for facilities	Wanted 1:1 care, Please take care of everything, Excessive demands	“They’re starting to demand a lot from our nursing homes for services for the older adult that they couldn’t actually do.”
Participation in emotional and physical care	Participation in care to understand the staff’s difficulties	Meal help, walk, Difficulties in changing diapers	“The last time family helped their mother with meals, they said that we had a lot of trouble. I think I can find out the difficulties and cooperate well.”
	Participation in care makes older adult more stable	Participation, stability, adaptation, positive,	“Occasionally, they come to stabilize their parents, and see if there is anything uncomfortable, and then the older adult naturally stay in a nursing home in a more peaceful state, and in this case, they can cooperate, and it is good for us.”
The role of family members present at the facility	Indifference to care after admission to the facility	Not coming, Even if the contact does not answer, Leave it alone	“Family comes often only for the first time, and if we contact an emergency, they won’t answer the phone. And sometimes they get rather angry. Why take we to the hospital … ”
	Transfer responsibility for care to facilities	Defer decision, Watcher role, Lack of interest, Non-cooperative on request	“I think that their role (family) has been transferred to a nursing home.”

#### Antecedents, consequences, and working definition of partnerships in LTC facilities

The antecedents of partnership examined via the literature review in the theoretical phase included “trust in institutional care” and “willingness to be involved as active participants.” Consequences of partnership affected family, staff members, and the residents. In other words, effects on family members included increases in empowerment in care and satisfaction with the nursing home and reductions in conflicts with staff (Bidmead & Cowley, 2005; Gallant et al., 2002; Hook, 2006). The effects on staff members included increase in job satisfaction, reductions in conflict and stress, and improvement in care quality (Bidmead & Cowley, 2005). The effects on residents included maintenance of well-being and health and improvement in the quality of life (Dupuis et al., 2016).

The working definition of partnership in LTC is to focus on a cooperative relationship that is an ongoing dynamic process. It also involves sharing of professional nursing knowledge, skills, and information regarding the patient’s condition as well as shared decision-making through appropriate role negotiation, the involvement of both parties in caregiving, and shared responsibility.

### Fieldwork phase

In the fieldwork phase, the dimensions and attributes of the partnership between staff members and residents’ family members in LTC facilities were identified via interviews. The staff members with seven attributes and family members with six attributes were identified.

The attributes identified via interviews with staff members included relationship building through communication, provision of information about the status of the resident, cooperative interaction in problem-solving, provision of high-quality care, coordination of role expectations at the facility, participation in emotional and physical care, and the role of family members present at the facility (Table III). The attributes identified via interviews with family members included mutual respect and equal relationships, seeking information about care, decision-making support, provision of care with dignity and consistency, recognition of care limitations at the facility, and care cooperation at the facility (Table IV).

**Table IV.** Category, Subcategory, and Codes Obtained Among Family Members in the Fieldwork Phase.

Category	Subcategory	Codes	A sample of participants’ statements
Mutual respect and equal relationships	Equal relationship to each other	Equality, relationship, Horizontal relationship	“I think it’s important for each other to think equally. We rather noticed that the employees didn’t think so.”
	A mutually respectful relationship	Mutual respect, understand, Thankful heart	“In my view, I am grateful for the caregiver to do what I can’t do at home, and I think this kind of attitude is important.”
Seeking information about Care	Timely information provided	Tell me what I need, Notify when problems occur	“Mom is not eating these days or she is telling me to come.” Thank you for saying something like this.
	Ask comfortably about the condition of the older adult	Comfortable atmosphere, Call anytime Welcome to call	It’s great that I can always call and ask about my mother’s condition.
Decision-making support	Tell me about the situation	Providing information on status, Provide the basis for judgement, Provide accurate information	“In order for the family to make a decision, they have to tell the status exactly. If you tell us the basis of judgment based on experience, we can do better judgment and it helps.”
	Help with judgement	Sharing experience, Give time, Encourage other family members to participate	Even if you know, it’s hard to make a decision. It would be much more helpful to tell us what to do and what others were like.
Provision of care with dignity and consistency	Consistent care	Care by the same staff at all times, Care by familiar staff, It is the same even if I visit unexpectedly	“I come here suddenly. They’re not surprised that I came suddenly, but they always do the same. The caregiver’s face is always bright … ”
	Care with dignity of the residents	Maintaining dignity, Privacy, Understand resident status	“My mom is a dementia patient. I am thankful to see that staffs are covering all the doors even when they dress my mom up. I didn’t do that at home … ”
Recognition of the limitations of care at the facility	Personalized care is difficult due to group life	Group life, Having a fixed time, Need to respect individual demands	“There’s a fixed time here. Since she has lived in a group, they have time to change diapers, so I’d like her to change it if it’s uncomfortable, but they can’t do it here. I understand. It’s a group life.”
	Can’t be like caring at home	Pretend not to know, Admit the situation Can’t be the same as my heart	“If it’s their best, there are some things you don’t like, but you have to pretend you don’t know. Wouldn’t be exactly the same as at home.”
Care coordination at the facility	Cooperate if requested.	Do not decline on request, Respond quickly on request, Don’t miss a call from the facility	“If you ask for cooperation, we’ll do it quickly, and that’s the best thing.”
	It’s good for each other to do what you’re told to do without taking action	Passive participation, Respect for the caregiving method, Do not take action	“I think participating in the care first might make the caregiver uncomfortable. From their point of view, they have their own way.”

### Final analytical phase

In the final analytical phase, the findings from the theoretical and fieldwork phases were analysed comprehensively to identify attributes and indicators of the partnership between staff members and residents’ family members in LTC facilities. The features of each of the seven attributes as identified in the literature review are presented in Table V.

**Table V.** Dimensions and Attributes of Partnership in Literature Review and Field Study.

Dimensions	Attributes in literature review	Attributes in field study	
		Staff	Family caregivers
Interpersonal factor	Relationship	Relationship building through communication	Mutual respect and equal relationships
	Information sharing	Provision of information about the status of the resident	Seeking information about care
	Shared decision-making	Cooperative interaction in problem solving	Decision-making support
	Professional competence	Provision of high-quality care	Provision of care with dignity and consistency
Environmental factor	Negotiation	Coordination of role and expectations at the facility	Recognition of the limitations of care at the facility
	Involvement in care	Participation in emotional and physical care	care cooperation at the facility
	Shared responsibility	The role of family members present at the facility	None

Ultimately, two dimensions (interpersonal factor and environmental factor), seven attributes, and 30 indicators were identified (Table VI). Interpersonal factor referred to the personal aspects of staff and family members and was classified into four attributes (relationship, information sharing, shared decision-making, and professional competence) with 20 indicators. Environmental factor referred to environmental or systematic aspects and was classified into three attributes (negotiation, involvement in care, and shared responsibility) with 10 indicators.

**Table VI.** Dimensions, Attributes, and Indicators of Partnership in Final Analytical Phase.

Dimension	Attributes	Indicators
Interpersonal factor	1. Relationship	1. Equal relationship 2. Mutually respectful relationship 3. Cooperative relationship 4. Mutual understanding and empathy 5. Open communication 6. Encouraging family members to visit facilities 7. Welcoming environment for family members’ facility visits
	2. Information sharing	8. Respect of other partner’s knowledge and care experience 9. Provision of information regarding the older adults before entering facilities 10. Provision of information regarding the older adults after entering facilities 11. Sharing of coping strategies
	3. Shared decision-making	12. Finding solutions together 13. Participation in the decision-making process 14. Decision-making support 15. Confidence in the information provided for decision-making
	4. Professional competence	16. Provision of safe care 17. Provision of care to maintain patients’ dignity 18. Provision of consistent care 19. Fulfilment of individuals’ special needs 20. Education regarding care provision
Environmental factor	5. Negotiation	21. Recognition of basic care in facility 22. Awareness of basic family roles 23. Respect for family’s needs 24. Discussion regarding role scope
	6. Involvement in care	25. Provision of opportunities to involve family in care 26. Positive support for family members involved in care 27. Appreciation of the value of caring
	7. Shared responsibility	28. Sharing common care-related goals 29. Common interest in the older adults’ condition 30. Active cooperation when requested

## Discussion

In this study, two dimensions (interpersonal and environment), seven attributes, and 30 indicators were identified for the partnership between staff members and residents’ family members in LTC facilities.

In 77% of previous studies, relationships have been identified as an attribute of partnerships (Hook, 2006). In this study, equal, mutual respectful and cooperative relationships were identified as major attributes of partnerships. Interpersonal relationship skills were identified as an antecedent (Wiggins, 2008). Communication, mutual understanding and empathy which were included as indicators in the present study were also chosen as attributes or subcategories in previous studies (Bidmead & Cowley, 2005; Choi & Bang, 2013; Dupuis et al., 2016). This is consistent with Choi and Bang (2013) posited that when partnership is perceived as an ongoing, dynamic process, its attributes and antecedents could be implied and duplicated. Additionally, encouraging family facility visits and creating a welcoming atmosphere during these visits have been found to positively affect the establishment of relationships with staff members and produce a feeling of trust regarding the safety and security of facility care. This is consistent with Bauer and Rhonda (2011) study indicating that a welcoming atmosphere for family visits played an important role in establishing constructive relationships, as it facilitated interaction between staff and family members.

Information sharing is an attribute based on the mutual perception that both staff and family members possess unique and valuable knowledge and experiences. Effective information sharing is an important resource in providing individualized care for older adults in LTC facilities and affects family members’ participation in care (Specht et al., 2000). In other words, since mutual respect and acceptance of family members as important caregiving resources strongly affect partnership building, it is necessary to educate staff and family members to improve their awareness. Therefore, plans for effective information sharing should be considered.

Professional competence refers to the provision of safe, consistent care while maintaining patients’ dignity, and previous studies have identified the concepts of professional knowledge and skills as attributes (Blue-Banning et al., 2004; Choi & Bang, 2013; Hook, 2006; Wiggins, 2008). Because the articles reviewed in the theoretical phase examined mainly acute hospital settings or children, knowledge and skills regarding diseases and treatment were emphasized; however, safety measures related to falls, a dignified end-of-life care, and the provision of consistent care were emphasized in the fieldwork phase. Specifically, the provision of consistent care referred to care services provided by a familiar person without frequent changes in caregivers. This is considered important in caring for older patients with dementia and could reflect the characteristics and culture of LTC facilities. Moreover, professional competence was included in the interpersonal domain in the present study; however, continuing education is required to enhance staff members’ competence (McWilliam et al., 2009) and should be supported in LTC facilities. This demonstrates that the two dimensions identified in this study were organically connected and supports the finding that partnership is an ongoing, dynamic process.

Shared responsibility refers to sharing common goals concerning care and a sincere interest in the patient’s status. In the fieldwork phase, “the role of family members exists at the facility” was identified as an attribute for staff members, but no attributes reflecting shared responsibility were observed for family members. Staff considered both staff and family members responsible for care, while family members considered only staff members to be responsible. This could explain family members’ lack of attributes for shared responsibility.

Despite partnership being a practical concept, previous studies have reviewed its concept in the literature. However, this study used a hybrid model that involving theoretical and fieldwork analyses, providing a concept of partnership that accounted for cultural differences in clinical practice. Therefore, the results of the study enhanced the understanding of partnerships from a nursing perspective. Moreover, the attributes identified in the study could be used in the development of tools to evaluate the partnership between staff members and residents’ family members in LTC facilities.

## Conclusion

The partnership between staff members and resident’s family members in LTC facilities is an ongoing, dynamic process involving the combination of interpersonal factor and environment factor. In other words, it could be defined as a cooperative relationship that involves sharing of professional nursing knowledge, skills, and information regarding the resident’s condition as well as shared decision-making through appropriate role negotiation, the involvement of both parties in caregiving, and shared responsibility.

The attributes identified in this study could be used in the development of tools to evaluate the partnership between staff members and residents’ family members in LTC facilities. In addition, the results could provide basic data for developing and assessing nursing interventions to enhance cooperative relationships.
